# What is the impact of miniscrew-assisted rapid palatal expansion on the midfacial soft tissues? A prospective three-dimensional stereophotogrammetry study

**DOI:** 10.1007/s00784-023-05154-4

**Published:** 2023-07-29

**Authors:** Laura L. Krijt, Aldin Kapetanović, Wouter J.L. Sijmons, Robin Bruggink, Frank Baan, Stefaan J. Bergé, René R.M. Noverraz, Tong Xi, Jan G.J.H. Schols

**Affiliations:** 1 Department of Dentistry - Orthodontics and Craniofacial Biology, Radboudumc Graduate School, Radboud University Medical Center, PO Box 9101, Dentistry 309, 6500 HB Nijmegen, the Netherlands; 2 Radboudumc 3D Lab, Radboudumc Graduate School, Radboud University Medical Center, PO Box 9101, 6500 HB Nijmegen, the Netherlands; 3 Department of Oral and Maxillofacial Surgery, Radboudumc Graduate School, Radboud University Medical Center, PO Box 9101, 6500 HB Nijmegen, the Netherlands

**Keywords:** Miniscrew-Assisted Rapid Palatal Expansion (MARPE), Facial soft tissue, Maxillary expansion, Three-dimensional stereophotogrammetry, Intra-oral scan, Distance map

## Abstract

**Objectives:**

To evaluate the midfacial soft tissue changes of the face in patients treated with miniscrew-assisted rapid palatal expansion (MARPE).

**Materials and methods:**

3D facial images and intra-oral scans (IOS) were obtained before expansion (T0), immediately after completion of expansion (T1), and 1 year after expansion (T2). The 3D images were superimposed and two 3D distance maps were generated to measure the midfacial soft tissue changes: immediate effects between timepoints T0 and T1 and overall effects between T0 and T2. Changes of the alar width were also measured and dental expansion was measured as the interpremolar width (IPW) on IOS.

**Results:**

Twenty-nine patients (22 women, 7 men, mean age 25.9 years) were enrolled. The soft tissue in the regions of the nose, left of philtrum, right of philtrum, and upper lip tubercle demonstrated a statistically significant anterior movement of 0.30 mm, 0.93 mm, 0.74 mm, and 0.81 mm, respectively (*p* < 0.01) immediately after expansion (T0–T1). These changes persisted as an overall effect (T0–T2). The alar width initially increased by 1.59 mm, and then decreased by 0.08 mm after 1 year, but this effect was not significant. The IPW increased by 4.58 mm and remained stable 1 year later. There was no significant correlation between the increase in IPW and alar width (*r* = 0.35, *p* = 0.06).

**Conclusions:**

Our findings indicate that MARPE results in significant but small changes of the soft tissue in the peri-oral and nasal regions. However, the clinical importance of these findings is limited.

**Clinical relevance:**

MARPE is an effective treatment modality to expand the maxilla, incurring only minimal and clinically insignificant changes to the midfacial soft tissues.

## Introduction

One of the primary aims of orthodontists while achieving ideal occlusion, and one of the main criteria by which patients judge the success of their own orthodontic treatment, is the improvement of facial harmony and esthetics [1, 2]. Previous research has shown that treatment for transverse maxillary deficiency, such as rapid palatal expansion (RPE) and surgically-assisted rapid palatal expansion (SARPE), impact the facial soft tissues and lead to an increase in nasal width and midfacial changes. This could be potentially undesirable [3–5].

Transverse maxillary deficiency is a common orthodontic problem, with a prevalence of approximately 10% in adults [6]. In children and young adolescents, it is effectively treated with RPE, while in older adolescents and in adults, SARPE is usually performed to open the increasingly interdigitated midpalatal suture, which requires higher forces, thereby avoiding dental side effects [7–9].

With the introduction of miniscrews in recent years, miniscrew-assisted rapid palatal expansion (MARPE) has progressively positioned itself as a non-surgical expansion therapy. MARPE appliances, such as the maxillary skeletal expander (MSE) or the Dutch Maxillary Expansion Device (D-MED), are tooth-bone-borne hybrid expanders using miniscrews to transmit the forces of the expansion screw into the palate in order to overcome the resistance of the midpalatal and circummaxillary sutures [10, 11]. MARPE was reported to be an effective and well-tolerated treatment in patients from the age of 16 onwards, with limited dental and periodontal side effects and a relatively short treatment duration [12, 13].

However, there has been limited research on the effects of MARPE on the facial soft tissues. A certain impact on the soft tissue is reported, but the available studies were mostly retrospective [14–16] and investigated short-term effects [15–17]. Furthermore, in order to accurately assess soft tissues, which are rounded and elastic, three-dimensional (3D) imaging techniques such as 3D stereophotogrammetry facilitate faster, non-invasive, and accurate facial scan images and facial measurements can be evaluated [18, 19].

The aim of the present clinical cohort study is to prospectively evaluate the impact of MARPE on the midfacial soft tissues in older adolescents and adults, both in the short and in the longer-term, using 3D stereophotogrammetry.

## Materials and methods

### Study design

A prospective clinical cohort study was set up at the Radboud University Medical Center, Department of Dentistry - Section of Orthodontics and Craniofacial Biology in Nijmegen, the Netherlands. Included were consecutive patients from the age of 16 onwards with transverse maxillary discrepancy who were treated with MARPE. The study included consecutive patients from the age of 16 onwards with a transverse maxillary discrepancy who were treated with MARPE. The transverse maxillary discrepancy was diagnosed clinically through an intra-oral examination, and was defined as a unilateral, bilateral or anticipated crossbite, or a discrepancy without constriction. 3D facial images and intra-oral scans (IOS) were made before expansion (T0), immediately after completion of expansion with MARPE (T1), and 1 year after the MARPE procedure (T2). Exclusion criteria were patients with cleft lip and palate, craniofacial anomalies or syndromes, or images of insufficient quality.

Approval from the Radboud University Medical Center Institutional Review Board (IRB no. 2019–6004) was obtained for this study and a written informed consent was obtained from all participants. The study was performed in accordance with the Declaration of Helsinki. All data were anonymized and de-identified prior to analyses.

### Treatment protocol

The Dutch Maxillary Expansion Device or D-MED (Radboudumc, Nijmegen, The Netherlands & Orthoproof, Nieuwegein, The Netherlands), an individualized, 3D designed and fabricated MARPE appliance based on an IOS, was used in the present study. It consists of a 3D printed stainless-steel structure including two bands around the upper first molars and four rigid connectors with circular screw holes for four miniscrews that connect the device to the palate. The technical aspects and clinical treatment protocol of the D-MED, including the step-by-step description of appliance placement, are described in detail in the study by Kapetanović et al. (2022), which was followed meticulously in the present study [10].

Maxillary expansion was initiated immediately after the insertion of the MARPE device. The screw was activated once a day, equivalent to 0.25 mm, and the expansion was closely monitored with weekly check-ups. The expansion was considered successful when the occlusal aspect of the palatal cusp of the upper first molars made contact with the occlusal aspect of the buccal cusp of the lower first molars, indicating the necessary amount of expansion had been achieved. Subsequently, the expansion screw was fixated (T1) and the appliance was left in place for 3 months to allow remodeling of bone in the separated midpalatal suture. After 3 months, the bands and connectors were removed and the treatment could be continued with a fixed straight-wire appliance, while the four miniscrews and the expansion screw were left in place for retention. They were removed either 12 months after termination of the active expansion or prior to surgery in case of a surgical orthognathic treatment (T2).

### 3D facial images used for soft tissue measurements

Acquisition of the 3D facial images was performed according to a standardized protocol with the 3dMDFace system (3dMD Ltd., Atlanta, GA, USA) with a two-pod configuration. Illumination was achieved using multiple LED light panels and system calibration was performed twice a day. Images were taken in natural head position by an experienced photographer. Patients were instructed to relax the facial muscles and have a neutral facial expression. 3D images of the patients were obtained at T0, T1, and T2. After obtaining the raw images, 3dMDPatient 4.0 (3dMD Ltd., Atlanta, GA, USA) was used to reconstruct the 3D images which were subsequently exported as a Wavefront OBJ file. The OBJ files were imported in 3DMedX® (v1.2.19.0, Radboudumc, Nijmegen, The Netherlands), a program for analyzing 3D images developed by the 3D Lab of the Radboudumc.

### 3D face orientation

**Fig. 1:**
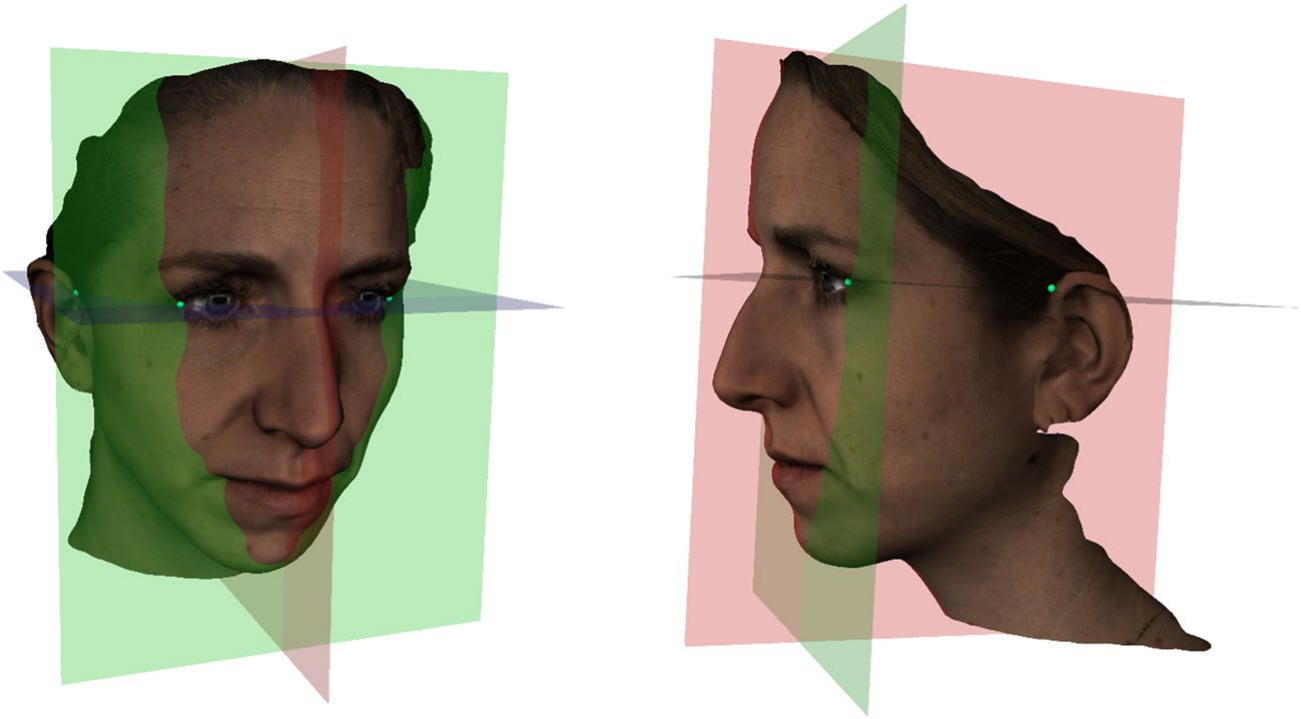
3D face orientation. The horizontal plane (purple), the coronal plane (green) and the sagittal plane (orange)

Firstly, the horizontal reference plane was defined as a plane dissecting both exocanthia, the points at the outer commissure of the eye fissure, and the average of both superaurale landmarks, the most superior points on the free margin of the auricle. Secondly, the coronal reference plane was defined as a plane dissecting through both exocanthia and perpendicular to the horizontal plane. Finally, the sagittal reference plane was defined as a plane dissecting the pupil reconstructed point (PRP) and perpendicular to the horizontal and coronal planes (see Fig. 1). The PRP, which served as the center point, was calculated by averaging both exocanthia.

### Landmarks

**Fig. 2:**
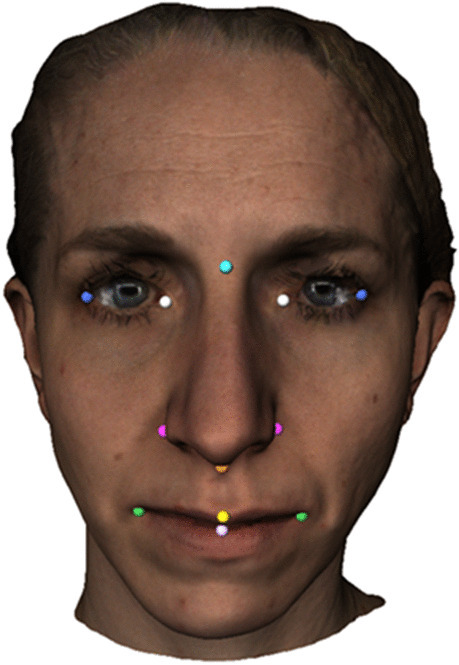
Landmarks

At T0, 12 landmarks were identified as described in Table 1 and shown in Fig. 2. These landmarks were used to define the contour of the five midfacial regions on each distance map.

**Table 1:**
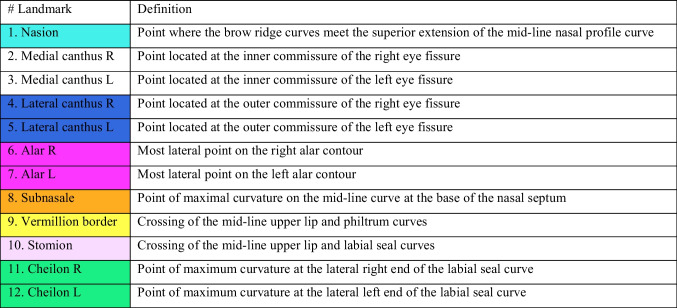
Landmarks. The colors correspond with the colored landmarks in fig. 2

### Matching

**Fig. 3:**
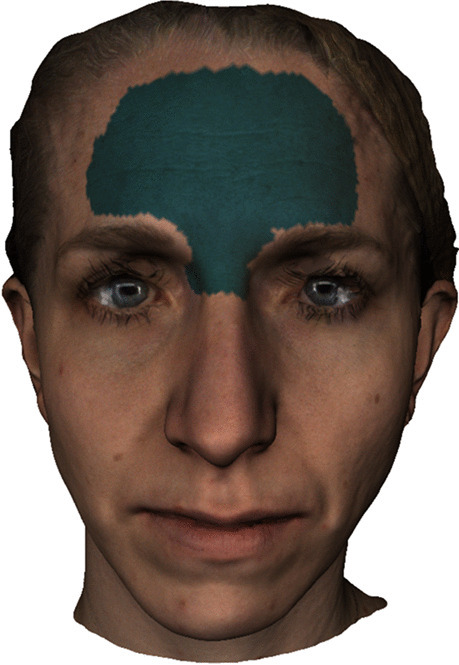
Stable structures

The 3D facial images were superimposed using a rigid iterative closest point (ICP) algorithm [20]. For superimposition, a stable region was used, defined by the forehead, the intercanthal region, and the soft tissue nasion region (see Fig. 3). This region was unaltered by the MARPE treatment [17].

### Distance maps

A distance map (DM) between a pair of superimposed 3D images was created to visualize the differences between the two 3D images. From each datapoint (vertices) on the 3D image of T0, perpendicular lines were extended until they reached T1 or T2. The length of each line indicates the local distance from T0 to T1 or T2. The inter-surface difference was visualized using a color-coded distance map in which red colors indicate a negative difference, or posterior movement, and green colors a positive difference, or anterior movement.

**Fig. 4:**
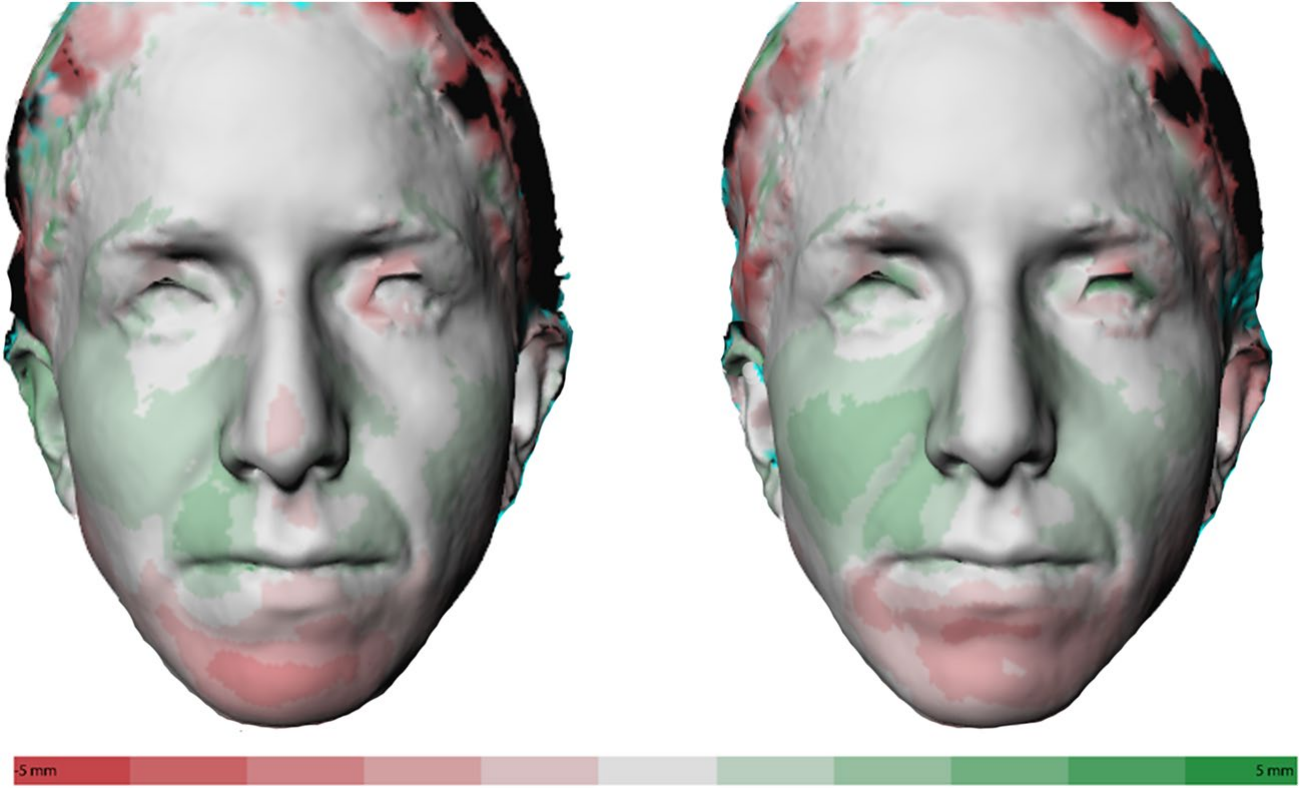
Distance maps. The differences between the two 3D images are visualized by color-coded distance maps. In the white areas low differences are found, which is to be expected on the forehead, the intercanthal region and the soft tissue nasion region, as these regions were used for superimposition. A green area shows a positive difference with a maximum of 5 mm for T1 or T2 in comparison with T0. A red area shows a negative difference with a maximum of 5 mm for T1 or T2 in comparison with T0. DM1, on the left, and DM2, on the right, are shown for the same patient

Two distance maps were generated: T0–T1 (DM1) to quantify the immediate effects and T0–T2 (DM2) to quantify the overall effects of MARPE (see Fig. 4).

### Measurements of regions

**Fig. 5:**
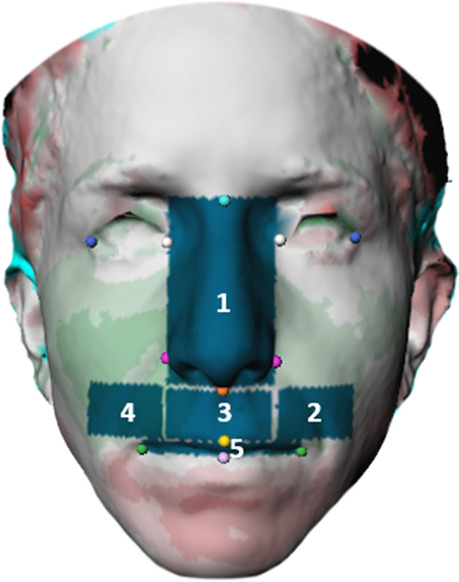
Five regions of the midfacial soft tissue

The 12 previously placed landmarks were used to define the contour of the five midfacial regions described in Table 2. The different areas of the distance maps could be selected and the deviations could be quantified based on the vertices of the triangles in the surface meshes that were included (see Fig. 5). On DM1 and DM2 the mean difference of each region was calculated.

**Table 2:**
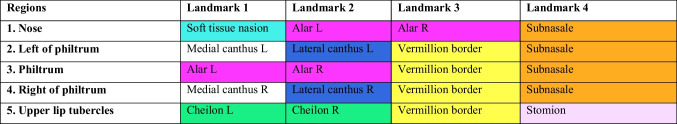
Five regions on midfacial soft tissue. The colors correspond with the colored landmarks in fig. 5

### Linear measurements

**Fig. 6:**
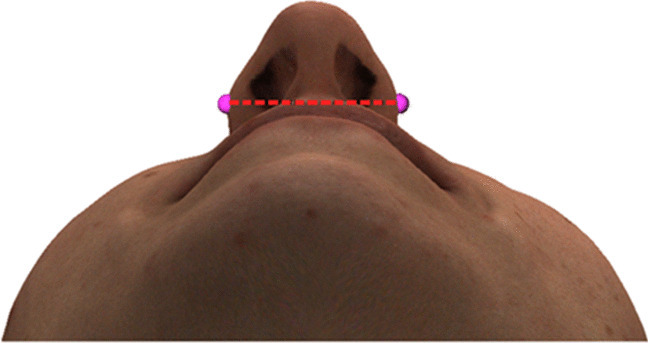
Alar width

Alar width was measured on the 3D image, defined as the distance on the 3D facial images between the alar left and alar right landmarks measured along the coronal plane at T0, T1, and T2 (see Fig. 6).

### Intra-oral scans used for intra-oral measurements

**Fig. 7:**
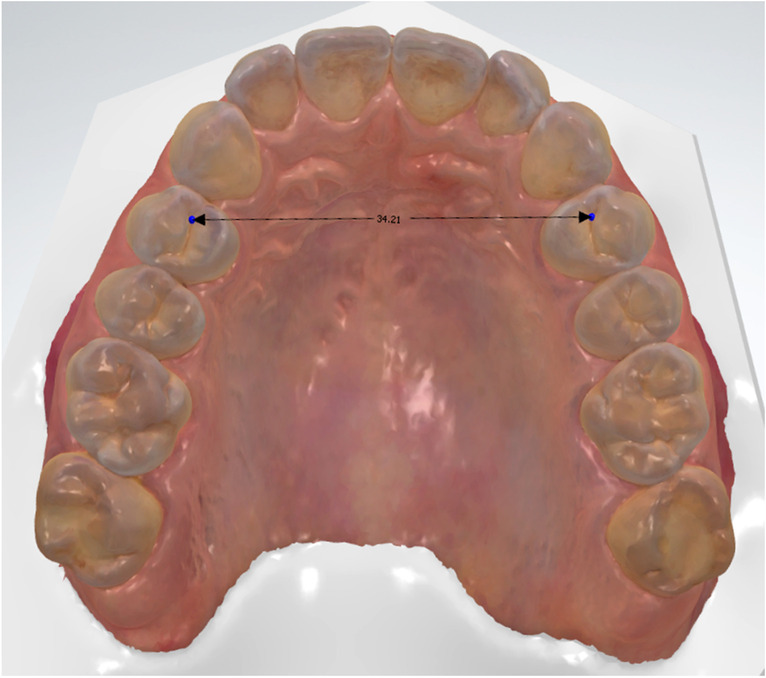
Interpremolar width

IOS were made by an experienced operator at T0, T1, and T2. The IOS were imported in Ortho Analyzer Orthodontics™ (v2021, 3shape, Copenhagen, Denmark) and the interpremolar width (IPW), defined as the transverse distance between the left and right maxillary first premolar mesial fossae, was measured on the three timepoints (see Fig. 7). The first premolar level was chosen for examining expansion as this location predominantly exhibits stable skeletal expansion (92%), hence providing a more accurate reflection of clinically significant expansion [10]. Furthermore, the premolars are the teeth that come closest to the alar base and for which data on expansion effect by MARPE (D-MED) were available.

### Statistics

Statistical analyses were performed using SPSS® Statistics version 25.0 (IBM Corp., Armonk, NY, USA). For intra-rater reliability, the process of matching and analyzing the distance maps was done twice for ten patients, with an interval of at least 4 weeks. For inter-rater reliability, ten patients were matched and analyzed by a second observer. To determine the inter- and intra-rater reliability a reliability analysis was performed using the intraclass correlation coefficient (ICC).

To test for differences in mean of outcome variables between T0, T1, and T2, analysis of variance with post-hoc testing with Bonferroni correction was used. Pearson’s correlation was used to determine the correlation between the IPW and the alar width. The level of significance was set at 0.05.

### Results

From 45 consecutively treated patients, 13 with insufficient scan data and three who stopped the treatment prematurely were excluded. Finally, 29 patients were enrolled, 22 women, and 7 men, with a mean age of 25.9 ± 7.8 years (range: 17.0–47.5 years). Post-hoc power analysis demonstrated a power of 0.88 for the main outcome variable based on a patient group of 29. The mean expansion duration was 35.7 days (T1) and the mean post-expansion duration was 12.6 months (T2).

### Intra-rater and inter-rater reliability

Intra-rater reliability of the superimposition of 3D images and analyses of distance maps proved to be excellent with an ICC of 0.97 (1^st^ measurements mean: 0.43, SD: 0.92, 2^nd^ measurements mean: 0.36, SD: 0.97).

Inter-rater reliability proved to be good with an ICC of 0.86 (2^nd^ observer mean: 0.44, SD: 0.95).

### Effects of MARPE on midfacial soft tissues

Statistically significant immediate soft tissue changes as the result of MARPE treatment (T0–T1) were found in four of the five midfacial regions: the nose 0.30 mm (SD: 0.55 mm, *p* < 0.01), left of philtrum 0.93 mm (SD:0.42 mm, *p* < 0.01), right of philtrum 0.74 mm (SD: 0.42 mm, *p* < 0.01) and the upper lip tubercles was 0.81 mm (SD: 0.87 mm, *p* < 0.01) (see Table 3). The mean soft tissue displacement in the anterior direction varied between 0.30 and 0.93 mm. In the 1-year follow-up period (T1–T2), the soft tissue in the philtrum region and left of philtrum region was displaced posteriorly by 0.59 mm (SD: 0.72 mm; *p* < 0.01) and 0.33 mm (SD: 0.62 mm; *p* < 0.01), respectively. As for overall effects between T0 and T2, statistically significant soft tissue changes in the anterior direction were found in four of the five midfacial regions: the nose 0.30 mm (SD:0.73 mm, p < 0.01), left of philtrum 0.60 mm (SD:0.57 mm, p < 0.01), right of philtrum 0.40 mm (SD:0.58 mm, p < 0.05) and the upper lip tubercles 0.71 mm (SD:1.06 mm, p < 0.01).

**Table 3:** Soft tissue outcomes. Mean difference (in mm), ± SD and 95% Confidence Interval of the difference (upper 95% CI; lower 95% CI) of the measurements of the five midfacial regions on DM 1 for immediate effects and on DM 2 for overall effects. To indicate the 1-year follow-up period T1–T2 was calculated. T-test was used for intergroup comparison between T0–T1 and T1–T2. Level of significance was set at 0.05

	T0–T1 (DM1)	T1–T2 (DM1-DM2)	T0–T2 (DM2)
Nose (mean 1)	0.30 ± 0.55 (0.23; 0.37)*	0.00 ± 0.21 (−0.08; 0.08)	0.30 ± 0.73 (0.20; 0.40)*
Left of philtrum (mean 2)	0.93 ± 0.42 (0.71; 1.16)*	−0.33 ± 0.62 (−0.10; −0.57)*	0.60 ± 0.57 (0.29; 0.91)*
Philtrum (mean 3)	0.21 ± 0.59 (−0.03; 0.45)	−0.59 ± 0.72 (−0.32; −0.87)*	−0.38 ± 0.76 (−0.77; 0.01)
Right of philtrum (mean 4)	0.74 ± 0.42 (0.50; 0.99)*	−0.34 ± 0.96 (0.03; −0.70)	0.40 ± 0.58 (0.00; 0.80)*
Upper lip TUBERCLES (mean 5)	0.81 ± 0.87 (0.44; 1.18)*	−0.09 ± 1.18 (0.35; −0.54)	0.71 ± 1.06 (0.20; 1.23)*

*DM:* distance map; *: *p* < 0.05

### Effects of MARPE on alar width

The mean alar width at T0, T1, and T2 were 32.18 mm (SD:2.76 mm), 33.77 mm (SD:2.75 mm) and 33.69 mm (SD:2.50 mm), respectively. The immediate effect of MARPE on the alar width (T0–T1) was an increase of 1.59 mm (SD:0.70; *p* = 0.08). Although the alar width decreased by 0.08 mm during 1-year follow-up, an overall increase of alar width between T0 and T2 was observed (mean 1.51 mm; SD:0.70 mm; *p* = 0.10).

### Effects of MARPE on IPW

The mean IPW at T0, T1, and T2 were 32.65 mm (SD: 2.90 mm), 37.23 mm (SD: 3.66 mm), and 37.29 mm (SD: 2.27 mm), respectively. The immediate effect (T0–T1) on the IPW was an increase of 4.58 mm (SD: 0.79 mm, *p* < 0.01). During the follow-up period, the IPW increased further (mean 0.07 mm; *p* = 1). The overall IPW expansion between T0 and T2 was 4.64 mm (SD: 0.79 mm, *p* < 0.01).

The Pearson correlation analysis demonstrated no statistically significant correlation between IPW and alar width changes during the expansion phase with MARPE (*r* = 0.35, *p* = 0.06). Furthermore, no correlation in the 1-year follow-up period was found (T1–T2) between the IPW and the alar width (*r* = 0.06, *p* = 0.76).

## Discussion

Even though MARPE has been reported to be highly successful, with increasing emphasis on facial appearance in recent years, some concerns persist and potential undesirable soft tissue changes could be an issue for patients and clinicians [2, 12, 17]. A limited number of studies has addressed this topic and, while the overall agreement was that the facial soft tissues showed notable positional changes after MARPE, there were differences with regards to the area and magnitude of the effects [14–17]. Lee et al. evaluated different landmarks around the nasal region and found that the nose tends to widen and move forward and downward, while Nguyen et al. found that both the cheeks and the nose showed lateral and forward movements whereby the nasal width increased by 2.05 mm [16, 17]. Abedini et al. reported a displacement in both the paranasal area and cheeks that remained stable after 1 year [14]. The alar width increase ranged from 0.93 to 2.05 mm [15–17]. However, the available studies were either retrospective [14–16], investigated only short-term effects [15–17] or only nasal soft tissues [17], included patients younger than 16 years whose soft tissues could still be developing [14] or used two-dimensional facial photographs [15], posing several limitations [21].

In the present study, both the short-term and longer-term, or overall, effects of MARPE on the midfacial soft tissues were prospectively evaluated. Given the rounded and elastic nature of soft tissue, stereophotogrammetry was considered the most adequate technique, being highly reliable and accurate and enabling the acquisition of 3D data without ionizing radiation exposure [22, 23]. The forehead, intercanthal region and soft tissue nasion were used as areas for superimposition since they were furthest away from the maxillary expansion area and could be considered the most stable [17]. Expansion with MARPE led to statistically significant positive changes, or anterior movements, in all defined soft tissue areas, except for the philtrum, both immediately (DM1) and overall (DM2), although the latter were somewhat smaller because of a limited amount of relapse. Conversely, the alar width increase was not significant.

In comparison, a systematic review and meta-analysis from Huang et al. on the soft tissue effects of RPE showed a statistically significant increase of nasal width, 0.84 mm, alar base width, 0.71 mm, and distance from the lower lips to Ricketts’ E-line, 0.75 mm, after expansion [3]. The soft tissues were evaluated on both 2D and 3D images, as well as by direct measurements. In a later study where a 3D facial scanner was used, 6 months after RPE, the increase of the nasal width and the nasal base width, 1.02 mm and 1.21 mm, respectively, were statistically significant, as well as the changes of the nose and upper lip, 0.55 ± 0.26 mm and 0.53 ± 0.67 mm, respectively [24]. These values were similar to those in the current study; however, the patients treated with RPE were growing, which is inherent to this therapy, thereby complicating comparison with the results for MARPE.

In this context, Truong et al. evaluated the effect of growth by comparing the nasal soft tissues of growing patients treated with RPE with a control group of patients around the same age who were not treated with RPE [25]. They found that, even though there was a significant increase of the nasal soft tissue immediately after expansion, it regressed to the mean of normal growth and development over time, representing a temporary stretching of the soft tissues rather than an actual and permanent soft tissue displacement [25].

SARPE, on the other hand, is performed in older adolescents and adults and changes after treatment cannot be related to growth. The soft tissue effects of SARPE have been studied extensively and the most recurring finding is widening of the nose after treatment, both in the short and in the longer-term, with an increase of the alar width ranging from 1.1 to 3.09 mm [4, 5, 26–30]. Furthermore, changes in the paranasal regions and an increased projection of the cheeks were often reported [5, 26, 31], whereas some studies also found a slight retro-positioning of the upper lip [31], a downward displacement of soft tissue pogonion [27] and increased facial convexity [26]. The absolute magnitude of these soft tissue changes was limited but potentially clinically relevant and the widening of the alars emphasized the perception of a more rounded nose [4, 5].

For MARPE, the overall soft tissue changes of the nose, the regions left and right of the philtrum and the upper lip tubercles observed in the present study were very small, ranging from 0.30 to 0.71 mm. Whether these effects were clinically relevant remains questionable, while the increase in alar width of 1.51 mm (SD:0.70 mm) was comparable to that found in studies on MARPE and SARPE. Given the transverse direction of expansion forces, some nasal widening could be expected, but it was not correlated with the amount of dental expansion. This suggests that the soft tissue changes were multifactorial and the magnitude of change could be influenced by other factors, such as the soft tissue thickness, tissue elasticity, or change in weight, particularly when evaluating longer-term effects.

The esthetic evaluation of these effects is challenging as it depends on the initial shape of the nose and the patient perception. It could be favorably perceived by patients with a narrow nose before treatment, whereas the opposite could be true for patients with a wider nose, but a threshold for the perception of nasal width has not been defined [32]. In addition to the quantitative measures used in this study, future research could also benefit from assessing the patient's perception of soft tissue changes. This could be done using before-and-after 3D photographs and assessing whether any changes are noticeable to the patients themselves, orthodontists, or laypeople. This could offer significant further insight into the impact and acceptance of MARPE treatment. It is known that unexpected changes in the facial appearance could be perceived as adverse effects. Clinicians should be aware of the effects of MARPE on the facial appearance and provide clear patient information before the start of treatment in order to manage patient expectations.

### Limitations

According to the treatment protocol, fixed appliances were placed 3 months after the end of expansion (T1). As a consequence, the results of T1–T2 and at T2 show both the change of the immediate soft tissue effects following MARPE and the effect of treatment with fixed appliances. However, this is inherent to any orthodontic expansion treatment.

Furthermore, the superimposition of 3D facial images was accurate only if the face was captured with the same facial expression at every timepoint. However, Maal et al. found a mean variation of 0.25 mm between 3D facial images [33]. In absolute terms, this is a very small variation, but given that most results in this study were under 1 mm, this could have impacted the outcomes.

## Conclusion

According to the present study, expansion with MARPE leads to minimal anterior movement of the nose, the regions left and right of the philtrum and the upper lip tubercles, immediately and as an overall effect. Furthermore, there is an increase in alar width.

These effects are statistically significant, with the exception of alar width, but are very small, and their clinical significance is limited. Overall, MARPE does not notably affect the midfacial soft tissues.
